# Reliability of the DIERS Formetric 4D Spine Shape Parameters in Adults without Postural Deformities

**DOI:** 10.1155/2020/1796247

**Published:** 2020-02-13

**Authors:** Brian F. Degenhardt, Zane Starks, Shalini Bhatia

**Affiliations:** ^1^A.T. Still Research Institute, A.T. Still University, Kirksville, MO 63501, USA; ^2^Department of Osteopathic Manipulative Medicine, A.T. Still University, Kirksville College of Osteopathic Medicine, Kirksville, MO 63501, USA; ^3^Department of Research Support, A.T. Still University, Kirksville, MO 63501, USA

## Abstract

The DIERS formetric 4D provides a safe method to monitor and track the progression of postural deformities over time. However, further evaluation of reliability is necessary. Reference values are also needed to indicate postural change. The current study examined the reliability of spine shape parameters produced by the formetric 4D in adults without postural abnormalities and established reference values to determine when real change occurs. Thirty participants were scanned during 1 week. Intraclass correlation coefficients (ICCs) were calculated for 40 spine shape parameters for scans with participants stationary between scans, scans with repositioning between scans, and between days. Within-day and between-day standard error of measurement (SEM), absolute relative SEM, and smallest detectable change (SDC) were reported. ICC for stationary scans was excellent for 29 parameters, good for 10 parameters, and fair for 1 parameter. With repositioning, ICC was excellent for 27 parameters, good for 12 parameters, and fair for 1 parameter. Between days, ICC was excellent for 26 parameters, good for 10 parameters, and fair for 4 parameters. Within-day SEM% was greater than 10% for 6 parameters. Within-day SDC ranged from 1.80 to 25.03 units for a single scan and from 0.97 to 17.93 units for 6 scans. Between-day SEM% was greater than 10% for 9 parameters. Between-day SDC ranged from 1.44 to 28.24 units for a single scan and from 1.05 to 22.2 units for 6 scans. Thirty-six of the 40 spine shape parameters from the DIERS formetric 4D reliably distinguished between participants over time. Reference values were established that can be used to track patient postural change over time. Future research should investigate the clinical relevance of these 40 spine shape parameters and determine when a clinically important change in posture occurs.

## 1. Introduction

Surface topography (ST) has been used on humans to produce 3-dimensional mapping of the body surface. One ST instrument, the formetric 4D (DIERS Medical Systems, Chicago, IL), shines parallel lines of light across the surface of the posterior trunk and, based on the distortion of those lines, reconstructs a digital image of the surface of the back and depicts a model of the vertebrae of the spine. This approach also produces unique postural measures based on skin surface reference points. Current standards for assessing posture are based on measurements from vertebral bony surfaces on spinal X-rays. A number of the formetric 4D measures have concurrent criterion validity with X-ray measures [1–6]. Other ST measures have been validated independently of X-ray comparison [7–9]. Because ST provides the ability to assess posture over time without the possible adverse effects from exposure to radiation, ST has been proposed as an alternative to X-ray. Because clinicians are familiar with managing patients using spinal X-rays, authors have suggested using both ST and X-ray for baseline measurements, then tracking posture more frequently with ST, and reassessing with X-ray when postural change is observed to reduce radiation exposure [2]. To characterize the clinical value of ST tracking of postural changes, ST measurements must be valid and reliable, and examiners need reference values to determine when an actual change has occurred to determine the clinical usefulness of these measures.

To demonstrate reliability, ST should be able to distinguish between participants in a population (i.e., high intraclass correlation coefficient [ICC]) [10–12] and exhibit agreement between repeated measurements of participants with stable posture (i.e., low standard error of measurement [SEM]). From SEM, the smallest detectable change (SDC) can be established to help clinicians determine whether a change in a measure is an actual change and not measurement error [11, 13]. The formetric 4D reports 40 named spine shape parameters. Evidence indicates that subsets of these parameters are reliable in healthy participants [8, 14–17] and in adolescents with idiopathic scoliosis [1, 2, 18, 19] when measurements are collected within a day [1, 2, 8, 14–20], over several days [14, 15, 17, 19], and by different technicians [16, 18, 19]. Of the 29 of 40 parameters previously studied, 21 were evaluated in only a single study [8, 17, 19], and only one study reported the SDC for 7 parameters [19]. Although most studies evaluating the reliability of the formetric 4D used ICC as their reliability index, the intended clinical value of the formetric 4D lies in its ability to track posture; therefore, SEM and SDC must be more thoroughly evaluated.

In the current study, we examined the reliability of all named spine shape parameters produced by the formetric 4D in adults without postural deformities using ICC and SEM, and determined SDC for each parameter to establish standards for monitoring postural change. We accounted for variability in posture attributable to moment-to-moment postural change associated with body sway and breathing, to the testing procedure, and to postural variability over 1 week.

## 2. Materials and Methods

Thirty participants, balanced between male and female and 3 body mass index (BMI) groups, were recruited from the local community through e-mail, posters, and word-of-mouth. Exclusion criteria included a history of spine surgery, back tattoos, or an inability to stand without assistance. The local institutional review board approved the study. All participants completed informed consent before participating.

**Figure 1:**
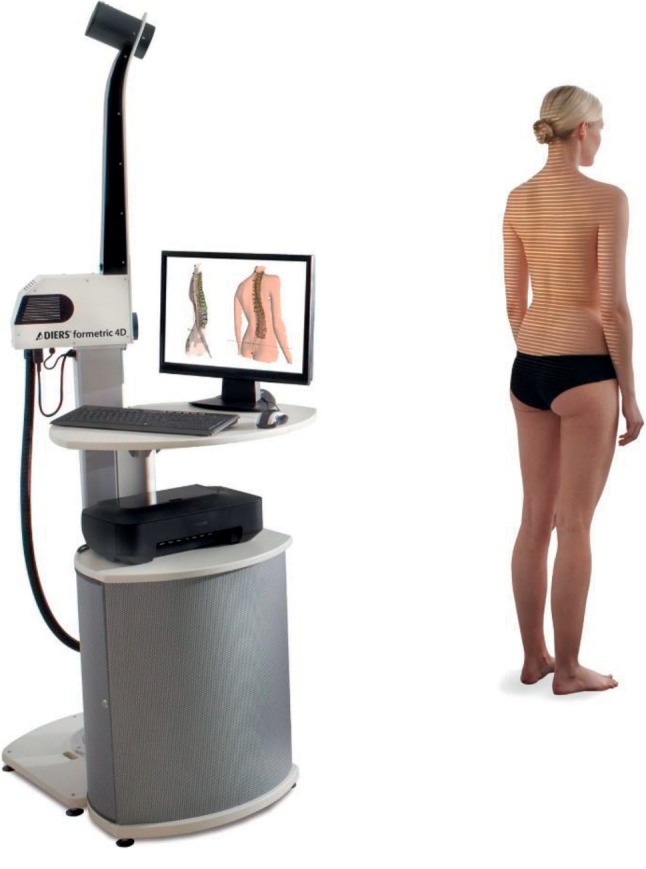
Example of formetric 4D projection unit set-up with participant positioning. Image used with permission from DIERS Medical Systems.

**Figure 2:**
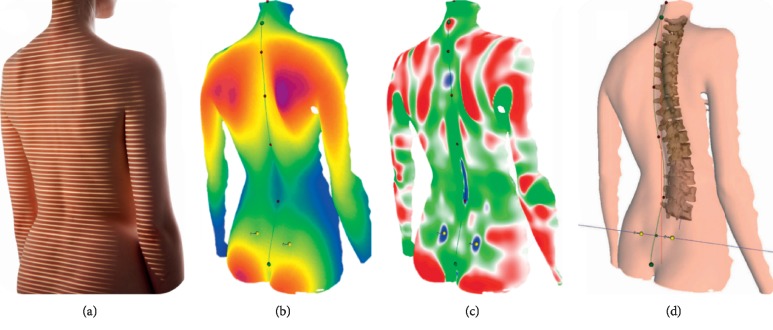
Formetric 4D recording (a), digitizing and 3D modeling (b), calculating curvature (c), and measurement procedure (d). Images used with permission from DIERS Medical Systems.

The formetric 4D scanning protocol performed in the current study was consistent with the manufacturer's guidelines and has been previously reported in detail [21]. Participants were positioned 2 meters from the formetric 4D projection and camera unit (Figure 1). The unit projected stripes of light on the surface of the participant's back (Figure 2(a)). Images of the surface of the back were recorded, digitized, and represented in 3D space (Figure 2(b)). Surface mean and Gaussian curvature were calculated (Figure 2(c)), and the underlying spine was rendered (Figure 2(d)). Each scan recorded 12-13 images over 6 seconds (2 Hz) and was processed based on the manufacturer's instructions. The instrument then produced 40 named spine shape parameters from a single image that is closest to the average position of the participant. Being unaware of the degree of measurement error this modality produces, the current study used data from each image to calculate the average for each scan and compared them to the manufacturer's reported values. Spine shape parameters were sorted into 5 subgroups (Table 1).

To determine the error attributed to moment-to-moment postural variability associated with body sway and breathing, participants were scanned 6 times within 6 minutes while they remained stationary between scans. Participants were scanned 6 times with repositioning (stepping off and on the evaluation platform) between scans to evaluate the impact of the set-up procedure on reliability. To determine the level of variability that occurs when posture can be assumed to be functionally stable, the stationary participant between-scan protocol was repeated on 5 days during 1 week within 1 hour of the same time as the original set of scans.

### 2.1. Statistical Analysis

Paired *t*-tests were used to test for any difference between the results from the single image closest to the average image and the calculated average from the 12 images. To test whether BMI affected the variability of parameters, within-participant variance was calculated and modelled for 3 BMI ranges (BMI1 = 20–24.9, BMI2 = 25–29.9, and BMI3 = 30–35).

Between-scan ICC for scans with stationary participants between scans (ICC-SBS) was calculated using a nested random effects model (model 1) built for each parameter using each image [21] from the 6 scans on each day. The ICC for scans with repositioning between scans (ICC-R) was calculated using another nested random effects model (model 2) for each parameter using each image from each of the 6 scans. To estimate between-day ICC (ICC-BD), variance estimates from model 1 were used. Parametric bootstrapping [22] was used to calculate the associated 95% confidence interval (CI) for each ICC. The Fleiss ICC (2, 1) formula was used to calculate ICC. ICC less than 0.40 indicated poor reliability, 0.4–0.59 indicated fair reliability, 0.6–0.74 indicated good reliability, and 0.75–1.0 indicated excellent reliability [23].

To measure actual change within the participant, SEM, SEM%, and SDC estimates were calculated. SEM% was calculated for parameters that report only positive or only negative values because parameters that cross zero in repeated scans bring the mean parameter value close to zero and disproportionately inflate SEM%. A value below 10% indicated high measurement stability [11, 24]. Using repositioning scans, within-day SEM% and SDC, which is the 95% CI of the change in parameter value [11], were calculated.

An additional set of random effects models (model 3) were built using data from the first scan from the first 4 days and all 6 repositioning scans. Between-day SEM, SEM%, and SDC were calculated for this model. Additional details about our models, ICC, SEM, and SDC calculations are available in Supplementary Materials [Supplementary-material supplementary-material-1]. All analyses were performed with SAS version 9.4 (SAS Institute, Inc., Cary, NC). A *P* value <0.05 was considered statistically significant.

## 3. Results

Thirty participants (white, age 30.1 ± 10.1 years, BMI 27.3 ± 4.6) completed the study. The dataset for 1 participant, a single stationary scan for another participant, and a single repositioning scan for a third participant were excluded because clothing obstructed the view of critical landmarks during data collection. In the final analysis, 1042 scans (13,547 images) were used from 15 males (age 31.9 ± 11.9 years, BMI 27.5 ± 4.2) and 14 females (age 28.1 ± 7.6 years, BMI 27.2 ± 5.1). For all parameters, no difference was found between mean parameter values produced from the single image closest to the average image selected by the formetric 4D and the calculated average from all 12 images within a scan (*P* > 0.05). The BMI1 and BMI3 groups each included 5 male and 5 female participants, while the BMI2 group included 5 male and 4 female participants. No difference was found in mean variability between the 3 BMI groups for all parameters (*P* > 0.05).

Of the 40 spine shape parameters, 29 had excellent reliability for ICC-SBS, 10 were good, and 1 was fair (Table 2). For ICC-R, 27 parameters had excellent reliability, 12 were good, and 1 was fair. For ICC-BD, 26 parameters had excellent reliability, 10 were good, and 4 were fair.

**Figure 3:**
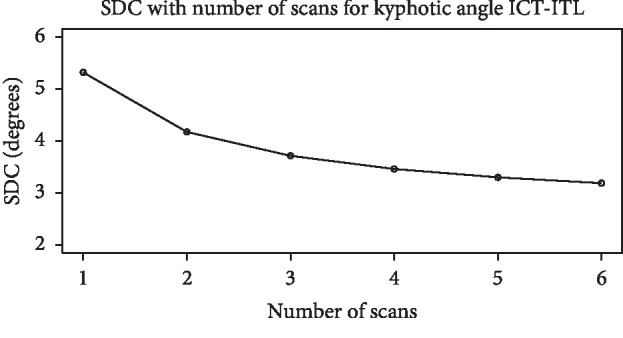
Smallest detectable change (SDC) for kyphotic angle ICT-ITL. Abbreviations: ICT: cervicothoracic transition point; ITL: thoracolumbar transition point.

Within-day SEM% and SDC were calculated from model 2 for a single scan (SDC_1_) and 6 scans (SDC_6_). Within-day SEM% was less than 10% for 19 of 25 parameters whose values did not cross zero (Table 3). Between-day SEM% and SDC were calculated from model 3. SEM% was less than 10% for 16 parameters (Table 3) and greater than 10% for 9 parameters. Within-day and between-day SDC_1_ and SDC_6_ are reported in Table 3. In both cases, SDC_6_ was smaller than SDC_1_ for each parameter. Variance estimates provided in Supplementary Materials [Supplementary-material supplementary-material-1] can be used to calculate SDC_2–5_.  Figure 3 presents an example of these variance estimates for the kyphotic angle.

## 4. Discussion

The current study was conducted to determine whether the 40 spine shape parameters of the DIERS formetric 4D can be used reliably in adults without postural deformity. We accounted for several sources of variability by analyzing data from each image (i.e., within scan), from scans during 1 day (stationary between scans and with repositioning), and from 5 days during 1 week to get precise estimates of variance to evaluate reliability. Mean and SD were reported for each parameter to describe the study cohort. ICC, SEM, and SEM% were used to evaluate the reliability of each of the parameters. We calculated within-day SDC to help clinicians know how to interpret parameter change when data is collected before and after interventions on the same day and calculated between-day SDC to evaluate postural change over longer periods of time. While we expected larger BMIs would increase variability, no significant differences in variability were observed up to a BMI of 35.0. Similar findings were reported for small subsets of parameters [8, 16, 20], but no study evaluated the full extent of the defined spine shape parameters as in the current study.

### 4.1. Reliability: Intraclass Correlation Coefficient

We estimated ICC to determine whether the formetric 4D can produce reliable data to distinguish between participants. To understand how variability from body sway and breathing influences reliability, ICC-SBS was calculated from scans where participants were stationary between scans. In previous studies where only a single image was collected per scan [8, 19] or a scan was reported as an average of images [17], the influence of body sway and breathing on reliability was minimized because within-scan variability was not fully accounted for. By using each image from each scan in the calculation of ICC, we accounted for all available within-scan variance. Overall, ICC-SBS was excellent (*n* = 29) to good (*n* = 10) for 39 of the 40 spine shape parameters, indicating moment-to-moment postural variability attributed to body sway and breathing has minimal influence on the measurement's reliability.

Concerned that repositioning the participant between consecutive scans could cause variability, we evaluated reliability when participants repositioned themselves between scans. Our ICC-R indicated repositioning had little effect on the reliability of the spine shape parameters. We found good agreement with previously reported parameters [8, 14, 17]. In a healthy population, only apical deviation left was reported to have fair ICC on a single day [14]; in the current study, apical deviation had good reliability. Because the previous study did not report their sample variance [14], the ability to make comparisons is limited. For 14 parameters that had not been previously reported in a population without postural deformities, the current study determined reliability ranged from excellent to good (0.96–0.61).

To determine the impact of normal day-to-day postural variability on reliability, ICC was used to evaluate data collected on 5 days during 1 week. Between days, 26 parameters had excellent reliability and 10 parameters had good reliability. These results supported previously reported excellent to good reliability between days for 19 spine shape parameters [14, 17]. The good reliability observed for pelvic torsion in the current study, however, was better than that of Guidetti et al. [14], where follow-up scans were completed on an unspecified separate day. In another study, scans were repeated the following day and the following week and demonstrated similar reliability to ours [17].

Four parameters (angle of coronal imbalance, vertebral rotation amplitude, trunk torsion, and vertebral rotation RMS (root mean square)) had fair ICC-BD in the current study but showed excellent to good reliability in previous studies [14, 17]. Reliability of the first 3 parameters decreased from good ICC-R to fair ICC-BD. Because these parameters had good ICC-SBS and ICC-R and the between-participant variability was constant, the decrease in reliability from ICC-R to ICC-BD can be attributed to incidental day-to-day postural variability that may be related to variations in fluctuating factors such as hydration, fatigue, or stress. Although these 3 parameters may provide useful information when repeated scans are completed during a single day, they were more susceptible to daily postural variability and should be used with caution to track posture over time.

Only vertebral rotation RMS had fair ICC in all 3 assessments. Vertebral rotation RMS represents the calculated RMS of the rotational amplitude along the entire spine. The fair ICC for this parameter may be explained by our population characteristics. Because ICC is more reflective of between-participant variance than within-participant measurement error [11], a population without postural deformities is more likely to have a smaller distribution of measurements (between-participant variance) than those with scoliosis or other postural deformities; thus, a lower ICC would be expected. Tabard-Fougere et al. [19] and Manca et al. [25] measured vertebral rotation RMS with excellent reliability in patients with adolescent idiopathic scoliosis; this higher reliability was likely because of the wider distribution of measurements in those populations (mean = 5.6 ± 2.6 and 6.8 ± 3.6, respectively). Only one other study reported ICC for this parameter in healthy participants [17], but because their sample had a wider distribution of measurements, they also reported higher reliability.

### 4.2. Measurement Error

In the current study, SEM was used to measure agreement between repeated scans to determine when spine shape parameters can be used to track change. SEM measures noise in repeated measurements from systematic and random error. A small SEM implies that the parameter has less variability and, therefore, is more reliable. Unlike ICC, SEM does not have specific reference values to indicate whether SEM is small enough to be clinically useful. Therefore, we used SEM% and SDC to evaluate the relative noise and to indicate when the actual change occurred.

SEM% was able to be calculated for 25 spine shape parameters. We used a threshold value of 10% to indicate high measurement stability [24]; however, this threshold is not universally accepted [11]. Within-day SEM% for 19 of the 25 parameters was below 10%, suggesting that these parameters may be most reliable, and thus most useful in detecting actual change over time. Six parameters in the spinal deviation subgroup had a within-day SEM% greater than 10%. These six parameters may not be precise enough to detect when a clinically meaningful postural change has occurred in a population without postural deformities. Between days, SEM% for 16 of the 25 parameters was below 10%. Three additional parameters to those listed above, flèche lombaire (depth of lumbar lordosis), flèche cervicale VP (depth of cervical lordosis at the vertebral prominens), and pelvic inclination appeared to be more susceptible to normal daily human variability. Only one other study considered these 4 parameters, but the authors used coefficient of variation, a similar statistic and threshold to SEM%, and reported values greater than 10% [17].

### 4.3. Clinical Applications

In general, clinicians can have confidence that the 4D formetric is reliable and that the sources of circumstantial error from breathing and postural sway are being accounted for in the standard processing performed by the instrument. So how should the current study's data be applied clinically? To determine if an immediate change in posture occurs after an intervention, the difference between preintervention and postintervention readings should be compared to within-day SDC_1_. The clinician should have confidence that any number greater than SDC_1_ indicates actual change in posture. Narrower SDC thresholds for all spine shape parameters can be easily achieved by doing repeated scans with the participant being repositioned between scans. This influence can be observed in the graphical example for kyphotic angle (Figure 3). Clinicians can use data in Supplementary Materials [Supplementary-material supplementary-material-1] for other spine shape parameters of specific interest to create their scanning standards, balancing scanning and processing time for repetitive scans to the value of increased SDC precision.

To track posture over time, between-day SDC_1_ should be used because it accounts for variability from normal day-to-day postural, instrument, and procedural factors. To our knowledge, only one study [19] has reported SDC for 7 spine shape parameters. Although our results found a smaller within-day and between-day SDC for 6 of the 7 parameters, that study [19] evaluated a cohort with scoliosis and used a single image of data in their analysis.

Overall, the DIERS 4D formetric is a relatively new system for evaluating posture with building evidence of the reliability of its 40 defined spine shape parameters. Several of these parameters, which are similar to measurements performed on postural spinal X-rays, have shown a reasonable level of concurrent criterion validity [1–6], with the formetric 4D reporting consistent but not identical values to X-rays. Because the remainder of the parameters measure something different from an X-ray, it is not possible to compare those measures with the clinical gold standard. These parameters have demonstrated criterion validity in comparison to models with calibrated surface measures. The current study addressed issues of reliability, a requirement of a valid measure, and defined the SDC of the spine shape parameters, thus allowing clinicians and researchers to know when change is not from measurement error. The next step is to determine how SDC relates to the construct of a clinically meaningful change. Determining the minimal clinically important difference (MCID)—the change in a parameter that is clinically important—is key to determining the value of integrating ST into standard medical care. Future research needs to evaluate SDC and patient-reported and functional measures for larger, more diverse populations over time, so reference values for both SDC and MCID can be established for relevant populations. Since the formetric 4D provides a safe, easily used, and objective measure of posture, these longitudinal studies can be easily performed, especially through multicenter collaborations.

### 4.4. Limitations

The current study had several limitations. Our sample population was narrow consisting of young to middle-aged adult participants with specific BMI ranges and no diagnosed postural deformities, so results can only be generalized to similar populations. In addition, data from only 29 participants were included in the final analysis. Studies have suggested including 15 to 50 participants in reliability studies [12, 26]. In our study, 36 repeated measurements on 29 participants allowed us to calculate precise variance estimates for reliability calculations [11] and were adequate for reliability testing. In the future, a larger and more diverse sample size would help to further refine reference values. Each subgroup in the analysis of the influence of BMI on variability included only 9-10 participants, but findings were similar to those previously reported. Further, one technician performed all scans so interobserver reliability could not be assessed although previous work has reported high interobserver reliability for the formetric 4D when automatic localization of landmarks occurred [3, 16, 18, 19].

## 5. Conclusions

The current study found that 36 of the 40 defined spine shape parameters from the DIERS formetric 4D can be used reliably to distinguish between participants during 1 week. Variability attributed to body sway and breathing was adequately accounted for by the study's protocol, and repositioning did not significantly affect reliability. Four parameters (vertebral rotation RMS, angle of coronal imbalance, vertebral rotation amplitude, and trunk torsion) appeared to be more susceptible to variability over time and, therefore, may not be appropriate for tracking postural change. Reference values to track postural change in participants without postural deformities were reported for each parameter using SDC.

## Figures and Tables

**Table 1:** Spinal shape parameters output by the formetric 4D and their definitions.

Parameter	Unit	Definition^1^
Distance Measurements		
Trunk length VP-DM	mm	The distance from VP to the center point between DL and DR (DM)
Trunk length VP-SP	mm	The distance from VP to the automatically localized sacral point (SP)
Trunk length VP-SP	%	The distance of VP-SP expressed as a percentage of VP-DM
Dimple distance DL-DR	mm	The distance from DL to DR
Dimple distance DL-DR	%	The distance of DL to DR expressed as a percentage of VP-DM
Trunk and Pelvis Imbalances		
Sagittal Imbalance VP-DM (trunk inclination)	°	The angle between the line connecting VP-DM and an external plumb line
Sagittal imbalance VP-DM (trunk inclination)	mm	The distance between VP and the connecting external plumb line
Coronal imbalance VP-DM (trunk imbalance)	°	The angle between the line connecting VP-DM and a plumb line through VP
Coronal imbalance VP-DM (trunk imbalance)	mm	The lateral distance between VP and DM
Pelvic obliquity (pelvic tilt)	°	The angle between the line connecting DL and DR and the horizontal
Pelvic obliquity (pelvic tilt)	mm	The difference in height between DL and DR
Pelvic torsion DL-DR	°	The torsion of the surface normals of DL and DR
Pelvic inclination (dimples)	°	The mean vertical components of the surface normals at DL and DR
Pelvis rotation (rotation correction [pelvis])	°	In the frontal plane, the angle of rotation of DR in relation to DL
Location of Postural Reference Points		
Inflection point ICT	mm	The point of maximum positive surface inclination above the kyphotic apex (KA)
Kyphotic apex KA	mm	The location of the posterior apex of the sagittal profile
Inflection point ITL	mm	The point of maximum negative surface inclination between the KA and the lordotic apex (LA)
Lordotic apex LA	mm	The location of the frontal apex of the sagittal profile in the lower region
Inflection point ILS	mm	The point of maximum positive surface inclination in the region between the LA and the sacrum
Flèche cervicale	mm	The horizontal distance between the cervical apex and the tangent through the KA
Flèche lombaire	mm	The horizontal distance between the LA and the tangent through the KA
Flèche cervicale (VP)	mm	The horizontal distance between the VP and the KA
Spinal Curve Angles		
Kyphotic angle ICT-ITL (max)	°	The angle between the surface tangents from the ICT and ITL
Kyphotic angle VP-ITL	°	The angle between the surface tangents from VP and ITL
Kyphotic angle VP-T12	°	The angle between the surface tangents on VP and the location of the calculated 12th thoracic vertebra (T12)
Lordotic angle ITL-ILS (max)	°	The angle between the surface tangents from ITL and ILS
Lordotic angle ITL-DM	°	The angle between the surface tangents from ITL and DM
Lordotic angle T12-DM	°	The angle between the surface tangents from T12 and DM
Pelvic inclination (symm.line)	°	The angle of the vertical surface normals from the horizontal of DM
Spinal Deviation		
Vertebral rotation (rms) (surface rotation)	°	The root mean square (RMS) of the horizontal components of the surface normals on the symmetry line
Vertebral rotation (max) (surface rotation)	°	The maximum value of the horizontal components of the surface normals on the symmetry line
Vertebral rotation (+max) (surface rotation)	°	The maximum value of the horizontal components of the surface normals on the symmetry line to the right
Vertebral rotation (-max) (surface rotation)	°	The maximum value of the horizontal components of the surface normals on the symmetry line to the left
Vertebral rotation (amplitude) (surface rotation)	°	The maximal spinal torsion calculated from the maximal rotation to the right and to the left
Trunk torsion	°	The maximal value of the horizontal components on VP compared to the horizontal components of the symmetry line on DM
Apical deviation VP-DM (rms) (lateral deviation)	mm	The RMS deviation of the midline of the spine from the direct connection VP-DM in the frontal plane
Apical deviation VP-DM (max) (lateral deviation)	mm	The maximum deviation of the midline of the spine from the direct connection VP-DM in the frontal plane
Apical deviation VP-DM (+max) (lateral deviation)	mm	The maximum deviation of the midline of the spine from the VP-DM line to the right
Apical deviation VP-DM (-max) (lateral deviation)	mm	The maximum deviation of the midline of the spine from the VP-DM line to the left
Apical deviation VP-DM (amplitude) (lateral deviation)	mm	The sum of the maximum deviation of the right and the left lateral deviation values

^1^Spine shape parameter definitions adapted from DIERS formetric III 4D Manual (Created 21.06.2010, Revision grade 5) and DIERS Optical Measurement of the Spine Information for the Assessment (Version 1, Created 04.08.2009). Abbreviations: DL: sacral dimple left; DR: sacral dimple right; ICT: cervicothoracic transition point; ILS: lumbosacral transition point; ITL: thoracolumbar transition point; VP: vertebral prominens.

**Table 2:** Intraclass correlation coefficients and 95% confidence intervals for scans completed with the participant in a stationary position between scans (ICC-SBS), with participant repositioning between scans (ICC-R), and between days (ICC-BD).

Spine shape parameter by subgroup	Intraclass correlation coefficient					
	ICC-SBS^1^		ICC-R^2^		ICC-BD^1^	
Distance measurements						
Trunk length VP-DM, (mm)	0.99	(0.98–0.99)	0.99	(0.98–1.00)	0.98	(0.96–0.99)
Trunk length VP-SP, mm	0.97	(0.96–0.98)	0.96	(0.93–0.98)	0.96	(0.93–0.97)
Dimple distance DL-DR, (%)	0.95	(0.91–0.97)	0.93	(0.87–0.95)	0.93	(0.89–0.96)
Dimple distance DL-DR, (mm)	0.92	(0.86–0.95)	0.89	(0.81–0.93)	0.90	(0.83–0.93)
Trunk length VP-SP, (%)	0.71	(0.60–0.80)	0.60	(0.41–0.73)	0.63	(0.48–0.74)
Trunk and pelvis imbalances						
Sagittal imbalance VP-DM, (°)	0.93	(0.89–0.96)	0.92	(0.85–0.95)	0.88	(0.80–0.92)
Sagittal imbalance VP-DM, (mm)	0.93	(0.89–0.96)	0.91	(0.84–0.95)	0.87	(0.80–0.92)
Pelvic inclination (dimples) (°)	0.93	(0.89–0.95)	0.92	(0.86–0.95)	0.85	(0.76–0.91)
Pelvic obliquity, (mm)	0.85	(0.77–0.90)	0.84	(0.73–0.90)	0.78	(0.66–0.85)
Pelvic obliquity (°)	0.83	(0.75–0.89)	0.82	(0.70–0.89)	0.76	(0.63–0.83)
Pelvic torsion DL-DR, (°)	0.74	(0.64–0.82)	0.68	(0.51–0.79)	0.60	(0.44–0.71)
Pelvis rotation, (°)	0.73	(0.63–0.81)	0.61	(0.42–0.74)	0.60	(0.44–0.71)
Coronal imbalance VP-DM, (mm)	0.72	(0.61–0.80)	0.69	(0.52–0.80)	0.60	(0.45–0.71)
Coronal imbalance VP-DM, (°)	0.71	(0.60–0.79)	0.67	(0.50–0.79)	0.59	(0.44–0.70)
Location of postural reference points						
Inflection point ILS, (mm)	0.97	(0.95–0.98)	0.97	(0.94–0.98)	0.95	(0.92–0.97)
Lordotic apex LA, (mm)	0.97	(0.95–0.98)	0.97	(0.93–0.98)	0.94	(0.90–0.96)
Flèche lombaire, (mm)	0.95	(0.92–0.97)	0.95	(0.91–0.97)	0.92	(0.86–0.95)
Kyphotic apex KA, (mm)	0.94	(0.91–0.96)	0.95	(0.90–0.97)	0.89	(0.82–0.93)
Flèche cervicale, (mm)	0.94	(0.90–0.96)	0.95	(0.91–0.97)	0.90	(0.83–0.93)
Flèche cervicale (VP), (mm)	0.92	(0.88–0.95)	0.93	(0.87–0.96)	0.86	(0.78–0.91)
Inflection point ITL, (mm)	0.93	(0.89–0.96)	0.92	(0.86–0.95)	0.91	(0.85–0.94)
Inflection point ICT, (mm)	0.82	(0.72–0.88)	0.84	(0.73–0.90)	0.76	(0.64–0.84)
Spinal curve angles						
Pelvic inclination (symm. line), (°)	0.96	(0.94–0.98)	0.97	(0.94–0.98)	0.92	(0.86–0.95)
Lordotic angle T12-DM, (°)	0.95	(0.92–0.97)	0.94	(0.89–0.96)	0.92	(0.86–0.95)
Lordotic angle ITL-DM, (°)	0.94	(0.91–0.96)	0.94	(0.89–0.96)	0.90	(0.84–0.94)
Kyphotic angle ICT-ITL (max), (°)	0.94	(0.90–0.96)	0.96	(0.92–0.97)	0.92	(0.86–0.95)
Lordotic angle ITL-ILS (max), (°)	0.92	(0.88–0.95)	0.91	(0.83–0.94)	0.87	(0.79–0.91)
Kyphotic angle VP-T12, (°)	0.91	(0.85–0.94)	0.96	(0.92–0.98)	0.89	(0.82–0.93)
Kyphotic angle VP-ITL, (°)	0.91	(0.86–0.94)	0.95	(0.91–0.97)	0.89	(0.82–0.93)
Spinal deviation						
Apical deviation VP-DM (max), (mm)	0.82	(0.73–0.88)	0.82	(0.70–0.89)	0.77	(0.66–0.85)
Apical deviation VP-DM (+max), (mm)	0.84	(0.76–0.90)	0.85	(0.74–0.90)	0.79	(0.68–0.86)
Apical deviation VP-DM (rms), (mm)	0.81	(0.72–0.87)	0.79	(0.65–0.86)	0.72	(0.59–0.81)
Apical deviation VP-DM (−max), (mm)	0.75	(0.65–0.83)	0.71	(0.55–0.81)	0.64	(0.50–0.75)
Apical deviation VP-DM (amplitude), (mm)	0.78	(0.68–0.85)	0.74	(0.59–0.84)	0.68	(0.54–0.78)
Vertebral rotation (max), (°)	0.73	(0.62–0.81)	0.69	(0.53–0.80)	0.68	(0.55–0.78)
Vertebral rotation (+max), (°)	0.71	(0.59–0.79)	0.66	(0.49–0.78)	0.62	(0.48–0.73)
Vertebral rotation (amplitude), (°)	0.71	(0.60–0.79)	0.73	(0.57–0.82)	0.59	(0.44–0.70)
Vertebral rotation (−max), (°)	0.71	(0.58–0.79)	0.69	(0.53–0.80)	0.65	(0.51–0.75)
Trunk torsion, (°)	0.63	(0.50–0.73)	0.67	(0.51–0.78)	0.56	(0.41–0.67)
Vertebral rotation (rms), (°)	0.53	(0.41–0.63)	0.49	(0.31–0.63)	0.42	(0.27–0.55)

^1^ICC-SBS and ICC-BD were calculated from the stationary between scans collected over 5 days. ^2^ICC-R was calculated from the repositioning scans collected on day 5. Abbreviations: DL: sacral dimple left; DM: middle point between DL and DR; DR, sacral dimple right; ICT: cervicothoracic transition point; ILS: lumbosacral transition point; ITL: thoracolumbar transition point; KA: kyphotic angle; LA: lordotic angle; rms: root mean square; SP: sacral point; VP: vertebral prominens.

**Table 3:** Within-day and between-day mean, standard deviation (SD), standard error of measurement (SEM), and smallest detectable change (SDC).

Spine shape parameter by subgroup	Mean	SD	Within-day^1^ (single scan)			Between-day^2^ (single scan)			Within-day (6 scans)			Between-day (6 scans)		
			SEM	SEM (%)^3^	SDC	SEM	SEM (%)	SDC	SEM	SEM (%)	SDC	SEM	SEM (%)	SDC
Distance measurements														
Trunk length VP-DM, (mm)	466.00	33.30	3.10	0.67	8.59	5.22	1.12	14.47	1.83	0.39	5.07	4.60	0.99	12.75
Trunk length VP-SP, (mm)	515.18	33.50	6.62	1.28	18.35	7.36	1.43	20.40	3.55	0.69	9.84	5.10	0.99	14.13
Trunk length VP-SP, (%)	110.56	1.42	1.32	1.19	3.66	1.17	1.06	3.24	0.68	0.62	1.88	0.65	0.59	1.80
Dimple distance DL-DR, (mm)	97.32	10.56	3.87	3.98	10.73	3.91	3.99	10.84	2.65	2.72	7.34	2.86	2.92	7.93
Dimple distance DL-DR, (%)	20.96	2.91	0.84	4.01	2.33	0.87	4.12	2.41	0.59	2.81	1.64	0.66	3.13	1.83
Trunk and pelvis imbalances														
Sagittal imbalance VP-DM, (°)	3.17	2.18	0.65	NA	1.80	0.76	NA	2.11	0.35	NA	0.97	0.53	NA	1.47
Sagittal imbalance VP-DM, (mm)	26.23	17.66	5.40	NA	14.97	6.36	NA	17.63	2.84	NA	7.87	4.43	NA	12.28
Coronal imbalance VP-DM, (°)	0.15	0.66	0.46	NA	1.27	0.52	NA	1.44	0.30	NA	0.83	0.38	NA	1.05
Coronal imbalance VP-DM, (mm)	1.29	5.62	3.85	NA	10.67	4.38	NA	12.14	2.45	NA	6.79	3.21	NA	8.90
Pelvic obliquity, (°)	−0.17	2.92	1.42	NA	3.94	1.67	NA	4.63	0.94	NA	2.61	1.28	NA	3.55
Pelvic obliquity, (mm)	−0.12	5.13	2.36	NA	6.54	2.73	NA	7.57	1.52	NA	4.21	2.04	NA	5.65
Pelvic torsion DL-DR, (°)	−0.07	1.95	1.55	NA	4.30	1.68	NA	4.66	0.92	NA	2.55	1.19	NA	3.30
Pelvic inclination (dimples), (°)	17.89	5.64	1.77	NA	4.91	2.41	NA	6.68	1.16	NA	3.21	2.01	NA	5.57
Pelvis rotation, (°)	−0.33	2.22	1.67	NA	4.63	1.61	NA	4.46	0.87	NA	2.41	0.81	NA	2.24
Location of postural reference points														
Inflection point ICT, (mm)	4.08	9.02	4.12	NA	11.42	4.53	NA	12.55	2.83	NA	7.84	3.42	NA	9.48
Kyphotic apex KA, (mm)	−190.25	23.24	5.45	2.86	15.10	7.95	4.18	22.03	3.33	1.75	9.23	6.65	3.50	18.43
Inflection point ITL, mm	−309.66	32.31	9.03	2.92	25.03	10.19	3.29	28.24	6.47	2.09	17.93	8.01	2.59	22.20
Lordotic apex LA, (mm)	−387.13	32.40	6.30	1.63	17.46	7.71	1.99	21.37	3.82	0.99	10.59	5.92	1.53	16.41
Inflection point ILS, (mm)	−462.73	42.02	7.44	1.61	20.62	9.12	1.97	25.28	5.07	1.10	14.05	7.45	1.61	20.65
Flèche cervicale, mm	74.42	16.49	3.85	5.17	10.67	5.84	7.98	16.19	1.92	2.58	5.32	4.83	6.60	13.39
Flèche lombaire, (mm)	37.53	12.18	2.84	7.57	7.87	4.29	11.40	11.89	1.53	4.08	4.24	3.58	9.52	9.92
Flèche cervicale (VP), (mm)	48.17	13.19	3.72	7.72	10.31	5.52	11.67	15.30	1.96	4.07	5.43	4.54	9.60	12.58
Spinal curve angles														
Kyphotic angle ICT-ITL (max), (°)	48.47	8.32	1.92	3.96	5.32	2.55	5.28	7.07	1.15	2.37	3.19	2.06	4.27	5.71
Kyphotic angle VP-ITL, (°)	46.66	7.91	1.86	3.99	5.15	2.65	5.69	7.34	1.12	2.40	3.10	2.21	4.75	6.12
Kyphotic angle VP-T12, (°)	43.10	7.59	1.64	3.81	4.55	2.35	5.47	6.51	0.99	2.30	2.74	1.96	4.57	5.43
Lordotic angle ITL-ILS (max), (°)	35.42	7.55	2.48	7.00	6.87	3.41	9.47	9.45	1.53	4.32	4.24	2.82	7.83	7.82
Lordotic angle ITL-DM, (°)	33.50	8.11	2.07	6.18	5.74	2.98	8.73	8.26	1.28	3.82	3.55	2.52	7.38	6.98
Lordotic angle T12-DM, (°)	29.94	8.39	2.13	7.11	5.90	2.98	9.76	8.26	1.30	4.34	3.60	2.47	8.09	6.85
Pelvic inclination (symm. line), (°)	19.68	7.27	1.47	7.47	4.07	2.43	12.10	6.73	0.98	4.98	2.72	2.16	10.76	5.99
Spinal deviation														
Vertebral rotation (rms), (°)	3.78	0.93	1.02	26.98	2.83	0.96	25.83	2.66	0.69	18.25	1.91	0.65	17.49	1.80
Vertebral rotation (max), (°)	1.98	6.02	3.98	NA	11.03	3.99	NA	11.06	2.80	NA	7.76	2.96	NA	8.20
Vertebral rotation (+max), (°)	5.68	2.79	2.10	NA	5.82	2.12	NA	5.88	1.45	NA	4.02	1.55	NA	4.30
Vertebral rotation (−max), (°)	−4.51	2.40	1.59	NA	4.41	1.58	NA	4.38	1.07	NA	2.97	1.13	NA	3.13
Vertebral rotation (amplitude), (°)	10.23	2.41	1.60	15.64	4.43	1.87	18.47	5.18	1.10	10.75	3.05	1.46	14.42	4.05
Trunk torsion, (°)	3.17	2.89	2.21	NA	6.12	2.37	NA	6.57	1.79	NA	4.96	1.98	NA	5.49
Apical deviation VP-DM (rms), (mm)	5.43	2.49	1.27	23.39	3.52	1.37	25.42	3.80	0.84	15.47	2.33	1.00	18.56	2.77
Apical deviation VP-DM (max), (mm)	4.26	8.69	4.01	NA	11.11	4.79	NA	13.28	2.64	NA	7.32	3.74	NA	10.37
Apical deviation VP-DM (+max), mm	8.04	5.13	2.10	26.12	5.82	2.34	29.43	6.49	1.44	17.91	3.99	1.81	22.77	5.02
Apical deviation VP-DM (−max), (mm)	−4.62	2.92	1.80	38.96	4.99	2.22	47.72	6.15	1.17	25.32	3.24	1.74	37.40	4.82
Apical deviation VP-DM (amplitude), (mm)	12.70	4.43	2.37	18.66	6.57	2.69	21.29	7.46	1.55	12.20	4.30	2.02	15.99	5.60

^1^Within-day SEM, SEM%, and SDC were calculated using repositioning data collected on day 5. ^2^Between-day SEM, SEM%, and SDC were calculated using the first scan from the first 4 days and the 6 repositioning scans collected on day 5. ^3^SEM% was calculated for spine shape parameters that report only positive or only negative values. Abbreviations: DL: sacral dimple left; DM: middle point between DL and DR; DR: sacral dimple right; ICT: cervicothoracic transition point; ILS: lumbosacral transition point; ITL: thoracolumbar transition point; KA: kyphotic angle; LA: lordotic angle; NA: not applicable; rms: root mean square; SP: sacral point; VP: vertebral prominens.

## Data Availability

The variance estimates used to support the findings of this study are included in the supplementary information file. The raw data used to support the findings of this study are available from the corresponding author upon request.

## Supplementary Materials

Supplementary Materialsor remove altogether Supplementary Material 1: additional description of the equations used for the statistical analysis. Supplementary Material 2: within-day and between-day  variance estimates for each spine shape parameter.
