# Supplementary material for: Reliability of the DIERS Formetric 4D Spine Shape Parameters in Adults without Postural Deformities
Source: Biomed Res Int. 2020 Feb 13;2020:1796247. doi: 10.1155/2020/1796247 (PMC7040381; doi:10.1155/2020/1796247)
Supplement: Supplementary Materialsor remove altogether — Supplementary Material 1: additional description of the equations used for the statistical analysis. Supplementary Material 2: within-day and between-day variance estimates for each spine shape parameter. [file 1796247.f1.pdf]

Supplementary Materials

Supplementary Materials 1. Additional description of the equations used for the statistical analysis

For each spine shape parameter, a nested random effects model (model 1) was built from data for all participants for all 5 days, using the initial 6 scans from each day and each of the 12 images within a scan as previously recommended [15]. Variance estimates were calculated as follows:

$$^{2} \quad = \sigma^2_{participants} + \sigma^2_{days} + \sigma^2_{scans} + \sigma^2_{residual} \qquad (1)$$

where $\sigma^2_{participants}$ was between-participants variance, σ^2_{days} was between-days variance, σ^2_{scans} was between-stationary scans variance, and $\sigma^2_{residual}$ was residual variance from images. Between-scan ICC (ICC-SBS) was calculated for stationary scans done on the same day.

For each spine shape parameter, another nested random effects model (model 2) was built using data from the 6 scans when participants were repositioned between scans and included all 13 images recorded within a scan. Total observed variance was calculated as follows:

$$\sigma^2_{total} = \sigma^2_{participants} + \sigma^2_{scans} + \sigma^2_{residual} \qquad (2)$$

The ICC for between scans with repositioning (ICC-R) was calculated from this model. To estimate between-day ICC (ICC-BD), variance estimates from model 1 were used.

To measure real change within the participant, SEM, SEM%, and SDC estimates were calculated for various scenarios. From model 2, within-day SEM was calculated for each parameter using all within-participant sources of variance as follows:

$$SEM = \sqrt{\frac{\sigma^2_{scans}}{n} + \sigma^2_{residual}} \qquad (3)$$

where *n* represented the number of follow-up scans. Within-day relative SEM (SEM%) was defined as follows:

$$SEM\% = \left|\frac{SEM}{mean} \times 100\right| \qquad (4)$$

The SDC for an individual, which is the 95% confidence interval of the change in parameter value [2], was calculated as follows:

$$SDC = 1.96 \times \sqrt{2} \times SEM \qquad (5)$$

Using repositioning scans, within-day SEM% and SDC was calculated for each follow-up scan as follows:

$$SDC_n = 1.96 \times \sqrt{2} \times \sqrt{\frac{\sigma^2_{scans}}{n} + \sigma^2_{residual}} \quad (6)$$

where *n* represented the number of scans.

An additional set of random effects models (model 3) were built using data from the first scan from each day and all 6 repositioning scans. In this case, variance estimates were calculated as follows:

$$\sigma^2_{total} = \sigma^2_{participants} + \sigma^2_{days} + \sigma^2_{scans} + \sigma^2_{residual} \quad (7)$$

Between-day SEM, SEM%, and SDC were calculated for this model as follows:

$$SEM_n = \sqrt{\frac{\sigma^2_{days}}{m} + \frac{\sigma^2_{scans}}{n} + \sigma^2_{residual}} \quad (8)$$

$$SDC_n = 1.96 \times \sqrt{2} \times \sqrt{\frac{\sigma^2_{days}}{m} + \frac{\sigma^2_{scans}}{n} + \sigma^2_{residual}} \quad (9)$$

where *m* represented the number of days and *n* represented the number of scans in a day.

Abbreviations: ICC, intraclass correlation coefficient; SDC, smallest detectable change; SEM, standard error of measurement.

Supplementary Material 2. Within-day and between day variance estimates for each spine shape parameter

Spine Shape Parameter	Within-Day Variance[1]			Between-Day Variance[2]			
	Patient	Scan	Residual	Patient	Day	Scan	Residual
Distance Measurements							
Trunk length VP-DM, mm	1147.000	7.488	2.093	1115.550	17.491	7.301	2.425
Trunk length VP-SP, mm	1140.920	37.491	6.377	1115.870	13.186	33.776	7.204
Trunk length VP-SP, %	2.588	1.534	0.213	2.373	0.000	1.145	0.227
Dimple distance DL-DR, mm	123.410	9.503	5.463	110.450	2.413	8.565	4.326
Dimple distance DL-DR, %	8.758	0.431	0.272	8.489	0.163	0.389	0.214
Trunk and Pelvis Imbalances							
Sagittal imbalance VP-DM, °	4.767	0.368	0.058	4.609	0.160	0.364	0.059
Sagittal imbalance VP-DM, mm	310.450	25.252	3.861	300.560	11.517	24.876	4.002
Coronal imbalance VP-DM, °	0.440	0.151	0.063	0.374	0.052	0.152	0.069
Coronal imbalance VP-DM, mm	32.516	10.574	4.245	26.971	3.940	10.670	4.598
Pelvic obliquity, °	9.404	1.359	0.658	8.862	0.657	1.385	0.755
Pelvic obliquity, mm	28.808	3.885	1.673	27.398	1.546	3.960	1.960
Pelvic torsion DL-DR, °	5.049	1.865	0.531	3.603	0.538	1.698	0.602
Pelvic inclination (dimples), °	36.033	2.144	0.977	29.853	2.774	2.088	0.931
Pelvis rotation, °	4.289	2.458	0.342	4.085	0.000	2.317	0.277
Location of Postural Reference Points							
Inflection point ICT, mm	88.583	10.809	6.189	89.953	3.433	10.623	6.468
Kyphotic apex KA, mm	543.220	22.377	7.335	522.890	31.716	22.874	8.651
Inflection point ITL, mm	938.210	47.489	33.992	970.310	23.700	47.634	32.595
Lordotic apex LA, mm	1095.230	30.070	9.609	1010.170	20.710	29.315	9.469
Inflection point ILS, mm	1826.400	35.553	19.769	1756.840	31.222	33.140	18.749
Flèche cervicale, mm	281.780	13.305	1.483	249.870	17.883	12.927	3.339
Flèche lombaire, mm	161.450	6.888	1.182	146.840	8.296	6.696	3.429
Flèche cervicale (VP), mm	175.280	11.946	1.857	151.280	14.849	11.758	3.818
Spinal Curve Angles							
Kyphotic angle ICT-ITL (max), °	79.892	2.836	0.851	73.320	2.663	2.703	1.147

Kyphotic angle VP-ITL, °	70.403	2.635	0.815	64.953	3.412	2.563	1.052
Kyphotic angle VP-T12, °	62.396	2.055	0.643	58.376	2.853	2.014	0.658
Lordotic angle ITL-ILS (max), °	60.066	4.577	1.565	60.371	5.439	4.381	1.810
Lordotic angle ITL-DM, °	68.231	3.192	1.103	67.014	4.296	3.092	1.518
Lordotic angle T12-DM, °	68.953	3.394	1.137	66.592	4.389	3.335	1.165
Pelvic inclination (symm.line), °	61.283	1.459	0.712	50.415	3.739	1.450	0.694
Spinal Deviation							
Vertebral rotation (rms), °	0.995	0.689	0.360	0.844	0.034	0.610	0.285
Vertebral rotation (max), °	35.807	9.572	6.260	32.274	1.065	8.536	6.300
Vertebral rotation (+max), °	8.653	2.766	1.653	6.931	0.519	2.511	1.455
Vertebral rotation (-max), °	5.694	1.640	0.874	5.085	0.233	1.471	0.807
Vertebral rotation (amplitude), °	6.849	1.606	0.952	6.053	1.004	1.618	0.866
Trunk torsion, °	10.064	2.010	2.873	8.003	0.861	1.990	2.743
Apical deviation VP-DM (rms), mm	5.930	1.093	0.526	5.440	0.303	1.061	0.526
Apical deviation VP-DM (max), mm	73.452	10.923	5.159	68.789	6.133	10.696	6.075
Apical deviation VP-DM (+max), mm	24.254	2.841	1.587	22.856	1.280	2.632	1.549
Apical deviation VP-DM (-max), mm	7.970	2.243	1.002	8.239	1.236	2.241	1.431
Apical deviation VP-DM (amplitude), mm	16.204	3.858	1.765	17.168	1.198	3.795	2.231

[1]Within-day SEM, SEM%, and SDC were calculated using repositioning data collected on day 5.
[2]Between-day SEM, SEM%, and SDC were calculated using the first scan from each of the first 4 days and the 6 repositioning scans collected on day 5.

Abbreviations: DL, sacral dimple left; DM, middle point between DL and DR; DR, sacral dimple right; ICT, cervicothoracic transition point; ILS, lumbosacral transition point; ITL, thoracolumbar transition point; KA, kyphotic angle; LA, lordotic angle; rms, root mean square; SP, sacral point; VP, vertebral prominens.
